# Supplementary material for: Meniscal and ligament modifications in spontaneous and post-traumatic mouse models of osteoarthritis
Source: Arthritis Res Ther. 2020 Jul 16;22:171. doi: 10.1186/s13075-020-02261-5 (PMC7364489; doi:10.1186/s13075-020-02261-5)

Supplementary Figure 1:

Control IgG immunostaining for collagen type 2 and SOX9 staining in both Str/ort mouse models and DMM joints. Col2 controls did not show unspecific staining in these sections, however SOX9 control showed some extracellular staining, which we can be confident is unspecific as SOX9 is a transcription factor therefore found in the nucleus only.


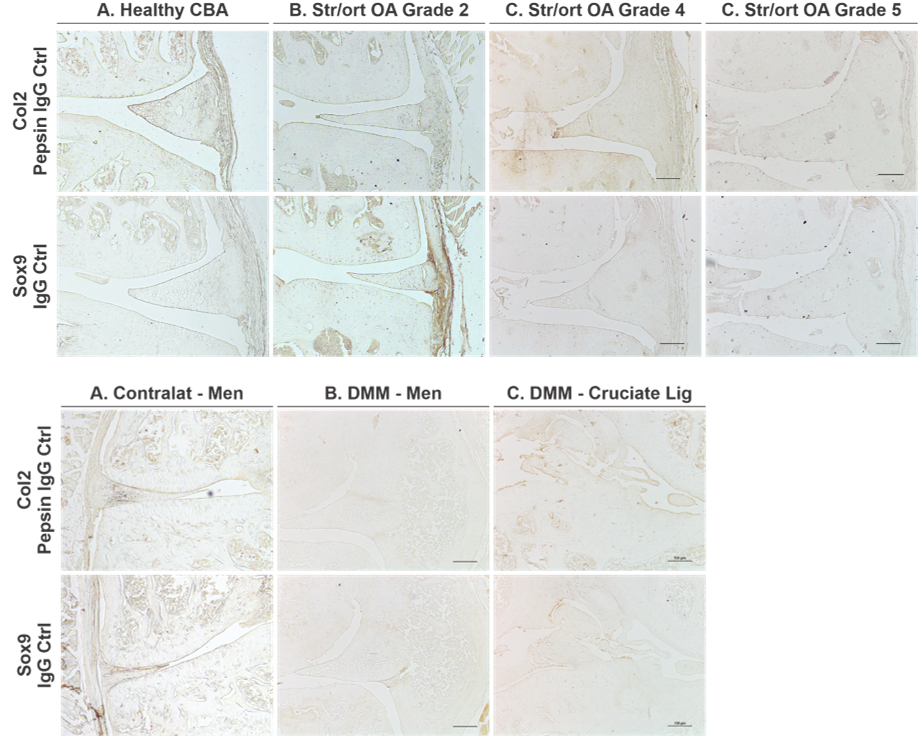

Supplement: Supplementary file 1 — Additional file 1. [file 13075_2020_2261_MOESM1_ESM.docx]
